# The role and therapeutic targeting of α-, β- and γ-secretase in Alzheimer's disease

**DOI:** 10.4155/fso.15.9

**Published:** 2015-11-01

**Authors:** Ruth MacLeod, Ellin-Kristina Hillert, Ryan T Cameron, George S Baillie

**Affiliations:** 1 Institute of Cardiovascular & Medical Sciences, College of Medical, Veterinary & Life Sciences, University of Glasgow, Glasgow G12 8QQ, UK

**Keywords:** alpha, Alzheimer's disease, amyloid hypothesis, beta, beta amyloid, gamma, secretase

## Abstract

Alzheimer's disease (AD) is the most common form of dementia in the elderly and its prevalence is set to increase rapidly in coming decades. However, there are as yet no available drugs that can halt or even stabilize disease progression. One of the main pathological features of AD is the presence in the brain of senile plaques mainly composed of aggregated β amyloid (Aβ), a derivative of the longer amyloid precursor protein (APP). The amyloid hypothesis proposes that the accumulation of Aβ within neural tissue is the initial event that triggers the disease. Here we review research efforts that have attempted to inhibit the generation of the Aβ peptide through modulation of the activity of the proteolytic secretases that act on APP and discuss whether this is a viable therapeutic strategy for treating AD.

## Abstract (lay abstract)

Alzheimer's disease (AD) is the most common form of dementia in the elderly but as yet there are no drugs that can halt the progression of this disease. In a theory called the ‘amyloid hypothesis’, researchers have proposed that the accumulation of a small protein fragment called beta amyloid or Aβ within brain tissue is the event which triggers Alzheimer's disease. Aβ is a derivative of the longer amyloid precursor protein (APP). Here we review research efforts that have attempted to inhibit the generation of Aβ through modulation of proteins called secretases which cut APP into Aβ.

Author edits made on: 20 May 2015.

**Figure 1.**
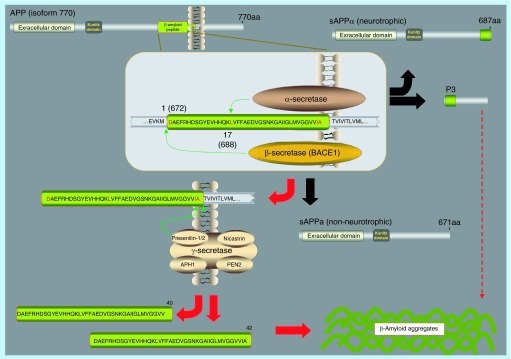
**Amyloid precursor protein processing.** The sequential proteolytic degradation of amyloid precursor proteins results in the generation of Aβ peptides, ultimately leading to the progression of Alzheimer's disease. Black arrows represent nonamyloidogenic processing of amyloid precursor protein and red arrows represent the amyloidogenic pathway.

**Figure 2.**
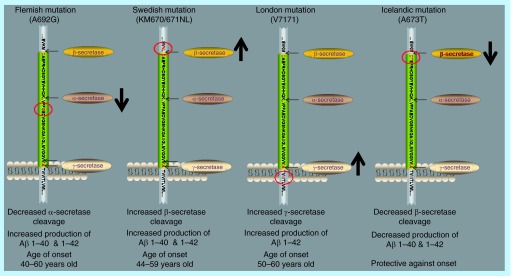
**Amyloid precursor protein mutations that alter amyloid precursor protein processing.** Examples of mutations in the amyloid precursor protein that alter its interactions with secretases, leading to altered production of Aβ peptides. The majority of mutations in the *APP* gene are pathogenic but the recently discovered Icelandic mutation confers a protective effect.

Alzheimer's disease (AD) is the most common form of dementia in the elderly and accounts for between 50 and 75% of all cases. By 2030 it is estimated that more than 65 million people will be living with dementia, with projections almost doubling every 20 years [1,2]. AD is a disorder characterized by the loss of neurons, mainly in the hippocampus and cerebral cortex [3]. The symptoms of the disease include erosion of memory and of other cognitive functions such as language and reasoning, as well as neuropsychiatric manifestations such as delusions and apathy [2,4]. In addition to its role in neurodegeneration, AD is the fourth most common cause of death in industrialized nations, preceded by cardiovascular disease, cancer and stroke [4]. The increasing burden of AD has resulted in intensive efforts by the scientific community to develop therapeutic agents to prevent the progression of the disease. However, as yet, there are no available drugs that can halt or even stabilize disease progression. Here we review research efforts that have attempted to inhibit the generation of the beta amyloid peptide through modulation of the activity of the proteolytic secretases that act on the amyloid precursor protein (APP) and discuss whether this is a viable therapeutic strategy for treating AD.

Alzheimer's disease was first described by Alois Alzheimer in 1907 [5]. Since then, its key pathological features have been shown to be senile plaques and neurofibrillary tangles, as well as ventricular dilation and atrophy of neural tissue [6]. The plaques’ main proteinaceous component is aggregated beta amyloid (Aβ), a derivative of the longer APP. The plaques are found primarily within the neocortex of the affected brain. Neurofibrillary tangles are accumulations of tau protein which are mainly located in or surrounding the hippocampus.

AD can be either familial, or sporadic. Familial cases of AD (FAD) have been traced back to mutations that alter the secretion of Aβ within the brain [7]. Similarly, other predisposing factors involved in sporadic AD have also been shown to affect Aβ. These results, together with the presence of Aβ within the senile plaques, have led to the formulation of the amyloid hypothesis as the leading model for AD [8]. The roles of α, β and γ-secretase play an essential part in this model.

## The amyloid hypothesis

The amyloid hypothesis was first proposed over 20 years ago [9]. It describes the accumulation of Aβ within neural tissue as the initial event that triggers the disease [8]. The accumulation is the result of an imbalance between Aβ production and clearance, which leads to the aggregation of Aβ and the formation of plaques which, in turn, cause the formation of neurofibrillary tangles [9]. The hypothesis was formed following two keystone observations: first, the identification of Aβ as the primary proteinaceous component of senile plaques [10] and, second, the identification of several mutations in familial AD that lead to the accumulation of Aβ [11–13].

Aβ is a derivative of the larger APP [14]. Aβ was first discovered in 1983 as part of senile plaques in patients with AD [15] and again in the brains of patients with Down's syndrome. This established the first link between AD and Down's syndrome and also led to the conclusion that the gene containing Aβ would have to be located on chromosome 21 [16]. APP was identified in 1987 as a ˜700 residue transmembrane protein. Since no other physiological function of this protein was known, except as the precursor to Aβ, it was simply designated APP. Its original function is still unclear. APP is expressed widely in normal human tissues including in the heart, lung, liver and skin [17]. The *APP* gene is located on chromosome 21, resulting in four-to-fivefold overexpression of APP in patients with trisomy 21 [18]. This explains the Aβ overproduction and early development of AD in individuals with Down's syndrome [14]. These results strongly implicated the deregulation of APP expression as a factor in AD development. APP, and all its related proteins, are single-pass transmembrane proteins with large extracellular domains. There are eight differently spliced isoforms of APP. The one most commonly expressed in the central nervous system (CNS) is the 695-residue isoform [19]. APP in itself is not neurotoxic, and does not produce Aβ until it undergoes proteolytic cleavage. It is produced in considerable quantities in neurons, where it is sorted via the Golgi and then shipped toward the axons [20]. APP can undergo cleavage in three different locations: at the N-terminal of the Aβ domain via β-secretase; at the C-terminal of the Aβ domain via γ-secretase; and within the Aβ domain via α-secretase. Proteolytic cleavage by α-secretase does not produce complete Aβ and, therefore, does not lead to the development of AD (Figure 1). Cleavage by β- and γ-secretase however yields several isoforms of the Aβ peptide that can aggregate into senile plaques [21].

Aβ itself is a 4-kDa peptide [22] that is primarily produced in neurons [23]. The Aβ domain in APP is located toward the C-terminal of the precursor protein [24], and is released extracellularly following cleavage by β and γ-secretase. The two most prevalent isoforms of Aβ are 40 and 42 residues in length. The two forms differ at the C terminal, where length is determined by the cleavage site of γ-secretase [25]. While Aβ40 is more common, the 42 residue isoform is the primary component of senile plaques as is it highly prone to aggregation [26,27]. Once released, Aβ will accumulate to form first oligomers, then fibrils and finally plaques of protein [28]. The amyloid plaques appear to then cause hyperphosphorylation of microtubule-associated tau protein. This causes the protein to accumulate and form neurofibrillary tangles, which cause synaptic dysfunction and contribute considerably to AD symptoms [29,30]. Thus, deposition of Aβ in neural tissue is a sufficient trigger event for the progression of Alzheimer's disease, according to the amyloid hypothesis.

In support of the hypothesis, a number of FAD associated missense mutations in APP have been characterized which induce changes in APP processing, resulting in increased Aβ production (Figure 2). The most notable of these are the KM670/671NL (Swedish) mutation and mutations at valine 717 including V717I (London) and V717F (Indiana) which frame the Aβ sequence [31–33]. The Swedish double mutation at the β-secretase cleavage site results in a six-to-eightfold increase in secreted Aβ peptide levels compared with wild-type APP. Other FAD associated APP mutations include: E693Q (Dutch); E693K (Italian), E693G (Arctic), D694N (Iowa) and A692G (Flemish) [31,33–38].

While the Flemish mutation induces a two-fold increase in Aβ_1–40_ and Aβ_1–42_ production due to reduced α-secretase activity (Figure 2), the Dutch and Iowa mutations do not alter levels of Aβ_1–40_ or Aβ_1–42_ relative to wild-type APP but are associated with accelerated fibril formation and increased pathogenicity from the resultant Aβ peptides [37,39–41]

While much is still left unanswered, for example, the physical connection between the formation of senile plaques and that of neurofibrillary tangles has not yet been explained, the amyloid hypothesis is supported by much experimental data and remains the most commonly accepted model for AD. Despite some drawbacks with the hypothesis, alternative hypotheses explaining pathogenesis of the disease have not been as robust. Therapies targeting Aβ amyloidogenesis have the theoretical potential to slow or even prevent further neurodegeneration, and the development of anti-Aβ therapeutics is regarded as a logical approach to the treatment of AD [9,42]. Here we look at the characterization of the secretases involved in APP processing and examine their ongoing therapeutic potential.

## α-secretase

The processing pathway of APP was long presumed to include a putative α-secretase – an enzyme that could cleave APP within the Aβ domain. This enzyme was known to be a metalloprotease that localized to the cell membrane and the Golgi complex. The α-secretase cleaves APP at residue L688, located in the middle of the Aβ domain (Figure 1). Cleavage through α-secretase can therefore not produce complete Aβ [43]. Instead it releases the soluble extracellular domain of APP (sAPPα) in a process called ‘ectodomain shedding’ [44]. This ectodomain is presumed to have neuroprotective properties [45]. The remaining membrane-bound C-terminal fragment of APP (C83) is then cleaved by γ-secretase to release a nontoxic p3 peptide [23]. Cleavage by α-secretase occurs in both a regulated and constitutive manner. Regulated cleavage occurs in the Golgi Complex under control of protein kinase C (PKC) [44,46], while constitutive cleavage occurs very rapidly at the cell membrane [44]. The first candidate enzyme for α-secretase was proposed in 1998 when ADAM17, also known as tumor necrosis factor-converting enzyme (TACE), was shown to have α-secretase activity, primarily within the Golgi Complex, that was regulated by PKC[47]. The existence of other α-secretases was presumed. In 1999 two more enzymes were shown to have α-secretase activity: ADAM9 and ADAM10 [44,48–49]. Like ADAM 17, both of them are part of the a-disintegrin and metalloprotease family (ADAM). All three of these enzymes have been confirmed as active α-secretases [49,50]. Initial knockdown of the candidate α-secretases seemed to demonstrate that release of sAPPα was never fully abolished, and initially it was concluded that the candidate α secretases showed significant functional overlap [49,51,52]. However, a more recent study showed that ADAM10, but not ADAM9 or 17, is essential for the constitutive α-secretase cleavage of APP and it has been concluded that ADAM10 is probably the most physiologically relevant α-secretase in neurons [51,53].

## Therapeutic potential of α-secretase

As α-secretase processing of APP involves cleavage within the Aβ peptide sequence, precluding Aβ formation, activation of APP a-secretase cleavage is considered an obvious potential treatment for AD [51]. Such activation would be presumed to both lower levels of Aβ and increase levels of the neuroprotective sAPPα [54].

Evidence for the rationale of such an approach comes from a study in which moderate overexpression of ADAM10 in an APP mouse model was shown to reduce levels of Aβ and prevented its deposition in plaques, as well as improving cognitive defects [54,55]. In addition, ADAM10 activity has been shown to be decreased in the platelet and spinal fluid of AD patients in accordance with disease progression [56,57]. Furthermore, a decrease in α-secretase has been demonstrated in sporadic AD brain samples [58].

Despite recent clarification of the role of ADAM10 in neurons, there is ongoing uncertainty over the potential effects of extensive activation of these proteases [59]. ADAM10 alone has been shown to cleave over 30 different substrates[54,60,61] and is thought to be widely expressed in non-neural tissues [62,63]. However, interestingly, studies have indicated that a number of the current drugs in use for treatment of AD can increase the activity of α-secretases via the activation of associated signaling cascades and it has so far been considered that this may represent the best therapeutic approach [52,64,65]. Selegiline, a selective monoamine oxidase inhibitor used to slow the progression of AD, has been shown to increase α-secretase activity via a protein trafficking related mechanism [66,67]. Atorvastatin, used to treat AD after it was shown that chronic statin use may be protective, also appears to induce activation of α-secretase [68,69].

A number of drugs intended as indirect α-secretase activators have progressed to the clinical trials stage for AD. The most prominent has been etazolate (EHT 0202), an GABA receptor modulator, which has reached Phase II human clinical trials after previously having been shown to stimulate sAPPα production and protect against Aβ induced toxicity in rat cortical neurons [70,71]. PRX-03140, a 5-HT4 agonist known to stimulate α-secretase, showed positive results in a Phase II trial in 2008 with improvement in cognition in AD patients, but no further studies have been announced [72]. NCT00951834 or epigallocatechin-gallate (EGCG), a polyphenolic compound from green tea, has also been shown to stimulate α-secretase via the PKC pathway and reduce cerebral amyloid deposition in AD mice [73–75]. Phase II/III trials investigating the benefit of this drug in AD are currently underway [73]. Finally bryostatin, a powerful PKC modulator known to increase the secretion of the α-secretase product (sAPPα), is currently undergoing Phase II trials for use in AD, with no results published to date [76].

## β-secretase

Akin to the situation for α-secretase, the involvement of β-secretase in APP processing has long been presumed. Several characteristics of this enzyme were known before the discovery of the enzyme itself. β-secretase was known to be widely expressed [77], but expression was most prominent in the pancreas and brain, especially in neurons [78,79]. Within cells it was expected to be localized to endosomes, lysosomes and the Golgi Complex [80,81] and to function optimally at an acidic pH [82].

In 1999 an enzyme which matched all these characteristics and showed proteolytic activity at the correct site on APP was discovered. The β-site APP Cleaving Enzyme (BACE1) was proposed as a likely candidate for β-secretase by four separate studies [24,83–85]. BACE1 is a transmembrane aspartic protease. It is about 500 residues in length with two active sites located on the lumenal side of the membrane. This allows the enzyme ready access to its substrate within the lumen of the Golgi, where it competes with α-secretase for APP, or within endosomes and lysosomes [24,26]. The putative β-secretase enzyme was known to be highly sequence specific [86,87], a characteristic that matches with BACE1, which cleaves APP at the N-terminal of the Aβ domain, either at residue Asp672 or Glu682 (Figure 1). This matches the most commonly found isoforms of Aβ [24]. As with α-secretase, cleavage of APP by BACE1 releases the soluble extracellular domain of APP (sAPPβ), however, the complete Aβ domain remains attached to the membrane-bound C-terminal fragment (C99) [23]. C99 is then cleaved by γ-secretase to release one of several Aβ isoforms, most commonly Aβ_1–40_ and Aβ_1–42_ [27].

BACE1 activity has been shown to be elevated in cases of sporadic AD [88,89]. However, β-secretase is not exclusively active in individuals with AD. Instead it appears to be producing Aβ during normal cell metabolism [77]. Therefore, the cause of AD is not simply the activation of β-secretase and its production of Aβ, but rather a change in the amount of APP processed by β-secretase. This could be due to mutations that reduce cleavage by α-secretase, favoring β-secretase instead, as can be seen in cases of familial AD [86,90] (Figure 2). It could also be caused by a shift toward differentially spliced APP, again favoring β-secretase processing, or simply due to an increase in APP expression [18].

## Therapeutic potential of β-secretase

The findings detailed above make BACE1 a primary target for drug research, as inhibition of the enzyme would effectively block Aβ production. Treatment of other diseases such as HIV using protease inhibitors had previously been successful and use of the same approach for BACE1 seemed promising [91]. *BACE1 -/-* homozygous knockout mice had originally displayed no adverse effects due to the absence of BACE1, which seemed to indicate that full inhibition may be tolerable [92,93]. *BACE1 -/-* mice fail to produce Aβ, do not develop amyloid plaques, and the knockout is able to rescue several phenotypes of AD-prone mice [94].

The past decade has therefore seen intense research efforts towards BACE1 inhibition. The crystal structure of BACE1 was initially used to develop the first generation of BACE1 peptidomimetic inhibitors [95]. These compounds seemed to show potent inhibitory activity and high selectivity [52]; however, as the active site of BACE1 is large, the required large molecular sizes also caused low membrane permeability across the blood–brain barrier (BBB) [95–97]. The development of smaller nonpeptidic inhibitors, with low enough molecular weights to allow for easier penetration of the BBB while still retaining a high affinity for the active site of BACE1 proved to be a long and difficult challenge. Achieving sufficient selectivity was another challenging issue, not least over BACE2, a BACE1 homolog with which it shares 64% identity [98–100]. Eventually such problems were overcome with the development of a small compound named CTS-21166 by CoMentis which possessed sufficient brain penetrability as well as high oral availability and selectivity. Phase I trials were conducted in 2008 and reductions in plasma Aβ levels of greater than 60% were announced, however no further studies have been planned for undisclosed reasons [101]. Eli Lily's LY2886721 initially showed reduction in plasma Aβ levels but had to be abandoned in July 2013 following Phase II trial due to liver toxicity [102]. Eisai's E2609 Phase I demonstrated a reduction in plasma Aβ levels as well as a dose-dependent reduction in Aβ CSF levels but Phase II trials have not yet been announced [103]. Development of TransTech Pharma's HPP854 and of Roche's RG7129 was terminated following Phase I trials for undisclosed reasons. Two BACE inhibitors have so far progressed to Phase II/III trial. Merck's MK-8931 began Phase II/III trial in November 2012 following successful reductions in the CSF levels of Aβ_1–40_ and Aβ_1–42_ during Phase II [104]. In November 2013, it was announced that the drug had passed initial safety evaluation and would progress to a second Phase III study. In September 2014, Astra Zeneca and Eli Lily announced the commencement of a large Phase II/III trial of Astra Zeneca's AZD3293 following claims of an encouraging safety profile and reduction in CSF levels of Aβ_1–40_ and Aβ_1–42_ during Phase I trial [105].

While these results have provided an encouraging proof-of-concept reduction in Aβ in humans following BACE1 inhibition, concerns over potential side effects from this approach have grown with time and increased understanding of the physiological role of this enzyme [106]. More recent animal studies seem to implicate BACE1 in the formation and maintenance of muscle spindle formation [107] and show cognitive and memory problems in homozygous knockout mice that were not apparent in previous studies [108,109]. *BACE1* knockout mice also display significantly higher mortality rates in their first weeks of life [110]. The progression of research into this enzyme has shown that BACE1 has a number of important alternative substrates, including proteins involved in myelination and sodium homeostasis [111,112]. This suggests a risk of morbidity with long-term BACE1 inhibition. The question may be whether the benefits of BACE1 inhibition outweigh the side effects in an elderly population. This has led to interesting speculation as to whether a partial rather than extensive inhibition of BACE1 activity may be enough to reduce Aβ levels [52,98]. Studies on mouse models seemed to indicate this might be a feasible approach. One study used heterozygous BACE1 gene knockout (*BACE1^+/−^*) mice to demonstrate that a 50% BACE1 reduction is sufficient to rescue deficits in brain function in an AD transgenic mouse model, 5XFAD[113]. A further study of *BACE1* heterozygote mice overexpressing mutant human APP (*PDAPP;BACE1+/−*) showed a considerable reduction in brain Aβ levels and plaque load as compared with *PDAPP;BACE1^+/+^* mice [114]. Heterozygous BACE1 knockout (*BACE1^+/−^*) mice appear to be healthy and do not demonstrate the phenotype shown the in more recent studies of *BACE1^-/-^* mice [109–110,112]. These data seemed to indicate that limited inhibition of BACE1 may represent a more viable approach. However, a more recent study showed that the phenotypic rescue and decreased Aβ burden associated with BACE1 haplo-insufficiency in the 5XFAD model may decline with age [115]. Therefore, further studies into the effects of partial suppression of BACE1 will be required to determine whether this is a feasible strategy.

The long wait for a successful, clinically beneficial inhibitor of BACE1 has caused many to question the validity of this protein as a target, and even to question the validity of the amyloid hypothesis itself [116,117]. Encouragingly, it was recently shown that a single residue mutation within the *APP* gene which reduces the ability of β-secretase to cleave APP confers a strongly protective effect against both AD and general cognitive decline in a human population [118], which many have taken as further support of the amyloid hypothesis and of BACE1 as a therapeutic target in AD.

## γ-secretase

The putative γ-secretase is an enzyme that cleaves the C-terminal fragment of APP following cleavage by either α or β-secretase to release the APP cytoplasmic domain [7]. Before its identity was confirmed, the protein was known to be fairly nonspecific in the sequences it would bind and cleave, producing divergent C-terminals in Aβ isoforms [7]. It was also known to be influenced by presenilin 1 and 2 (PS-1 and PS-2), which appeared to determine where at the Aβ C-terminal γ-secretase would cleave the protein.

The putative γ-secretase enzyme constituted a riddle in earlier years, as it appeared to be cleaving the APP C-terminal fragment within the hydrophobic environment of the plasma membrane [7]. It was finally shown to be a protein complex, and a founding member of a new class of intramembrane-cleaving proteases [119]. In this protein complex, presenilin represents the active site after being cleaved into an N- and a C-terminal fragment that associates into a heterodimer [120] with aspartic protease activity [121]. The proteins nicastrin (NCSTN) [122,123], anterior pharynx-defective 1 (APH-1) [124] and presenilin enhancer 2 (PEN-2) [125] were also found to associate with the presenilins to form the γ-secretase complex (Figure 1). The components of the γ-secretase complex are widely expressed in a variety of tissues out with the brain including liver, heart and lung [126,127]. All of the presenilin-associated proteins are embedded in the membrane [123–125]. The substrate passes in between the two presenilin fragments which appear to form a hydrophilic pocket in the membrane that allows for the proteolytic cleavage to occur [128,129]. Presenilin mutations appear to be the main cause of familial AD with more than 150 causative mutations having been mapped to the genes encoding the presenilin proteins, PSEN1 and 2 [130,131]. This is suggestive of a key role for the γ-secretase complex, at least in the familial form of AD. Most such mutations appear to increase production of Aβ_1–42_ over Aβ_1–40_ by promoting cleavage at residue 639 of APP over residue 637 [132].

## Therapeutic potential of γ-secretase

As with β-secretase, inhibition of γ-secretase represents an obvious logical strategy for inhibiting the generation of Aβ. Notwithstanding its status as a protein complex, the development of potent γ-secretase inhibitors able to cross the blood–brain barrier proved fairly simple [133,134,135]. However, γ-secretase had been shown to cleave a wide range of substrates, carrying out a much broader function than originally anticipated [136,137]. By far the most significant of these substrates is Notch, a cell surface signalling receptor that is essential for many aspects of cell development and differentiation [138,139,140,141], and which may also play a role in tumour suppression [142]. For these reasons, potential problems with toxicity in γ-secretase inhibition had long been predicted [52].

Indeed thus far clinical trials of γ-secretase inhibitors have demonstrated significant adverse effects as well as lack of positive effect on cognition [143]. γ-secretase has been the most commonly studied anti-amyloid target in clinical trials but as yet no improvement in cognitive function or slowing of cognitive decline has been confirmed [144]. In a recent high profile failure, Eli Lily's semagacestat (LY450139) was discontinued after two Phase III trials demonstrated not only failure to slow disease progression but also significant cognitive worsening [145]. An increased incidence of skin cancer was also reported, most likely due to concomitant Notch inhibition. Development of Bristol-Myers-Squibb's avagacestat (BMS708163) was halted in November 2012 after Phase II trials, again due to lack of positive effect on cognition, worsening cognition and increased risk of skin cancer [143,146].

As with β-secretase inhibition, such high profile, disappointing results for inhibition of γ-secretase have prompted serious questions about the feasibility of therapeutically targeting this complex as an AD strategy and have also prompted considerable debate over the long and expensive pursuit of the amyloid hypothesis as the central dogma in AD research [147].

The future of γ-secretase inhibition as an AD treatment strategy may depend on two newer strategies: first, the successful development of APP selective ‘Notch-sparing’ γ-secretase inhibitor compounds, capable of lowering Aβ levels without inhibiting the processing of Notch [148]; second, the development of γ-secretase modulators, which aim to cause a shift from Aβ_1–42_ species toward the shorter and less pathogenic forms of Aβ, while also sparing Notch [149].

Results so far have not been encouraging. Avagacestat was initially thought to act as a Notch-sparing γ-secretase inhibitor but later studies showed this to be false [150]. A clinical trial for the Notch-sparing inhibitor ELND006 (Elan Corporation) was halted in 2010 due to liver toxicity [151]. A second Notch-sparing inhibitor, Begacestat (GSI-953) demonstrated lowered Aβ levels in the CSF during Phase I trial but development was discontinued in late 2010 [152]. The γ-secretase modulator Tarenflurbil showed positive results on cognition in Phase II but was terminated after Phase III trials due to poor results [153–155]. Chiesi's γ-secretase modulator CHF-5074 reached Phase II trials but was halted for undisclosed reasons [156]. A further γ-secretase modulator, NIC5–15, is in Phase II trials with results yet to be announced [157]. Despite the poor results with earlier drugs hope still remains around this modulatory approach.

## Conclusion

From the information reviewed here it remains far from certain whether targeting the secretases involved in APP processing will yield the ground breaking therapeutic that is urgently required to treat AD. The number of high-profile failures in recent years has severely impacted the confidence of large pharmaceutical companies in the continuation of research and development programs in the neuroscience area and a number of companies have scaled back their risk in this field. Nonetheless, the potential rewards for discovering a drug to treat AD prevent a full retreat by key players.

Pharmacological attempts to target Aβ clearance through mechanisms to induce active or passive immunity have produced similar negative results leading to more recent high profile clinical failures, namely Bapineuzumab, developed by Johnson & Johnson and Pfizer [158] and Solanezumab, developed by Eli Lilly [159], both of which failed their primary end points in Phase III.

The amyloid hypothesis has now been the mainstay of therapeutic research in Alzheimer's disease for over two decades. The series of high profile clinical failures has inevitably called into question the viability of the hypothesis itself. A number of issues have plagued the amyloid hypothesis since its inception. First, the level of Aβ burden does not often correlate with clinical manifestation of the disease. In several studies amyloid plaques were apparent in control samples from humans despite no evidence of cognitive decline [160–163]. However, other investigations have found a much stronger correlation between levels of soluble Aβ oligomers and severity of cognitive decline [164,165].

Second, the difficulty in isolating the specific neurotoxic species of Aβ and characterizing its effects makes research problematic. Early studies demonstrated that aggregation of Aβ is essential for the cytotoxic effects of Aβ [166] but it was also noted that different preparations result in different potencies of the Aβ peptide [167]. Furthermore, soluble intermediate species of synthetic Aβ are made up of several distinct conformations which appear to have differential neurotoxic effects on cultured neurons. These include: oligomers composed of 15–20 monomers; small diffusible Aβ oligomers known as ADDLs (Aβ-derived diffusible ligands); and protofibrils (strings of oligomers) [168–171].

Further criticism of the evidence underpinning the amyloid hypothesis revolves around the current transgenic mouse models of AD, which do not fully recapitulate the disease. Despite increased Aβ deposition in these models, there appears to be a lack of coincidental neuronal loss. This is thought to be due mainly to species differences in neuronal susceptibility to Aβ accumulation, a lack of the human tau protein in mice, as well as the lack of a human-like inflammatory response which also plays a pivotal role in the progression of the disease [9].

Critics of the primacy of the amyloid hypothesis in AD research have argued that a simplistic focus on this one approach may have diverted attention from other important associations in AD. Some argue that while APP processing is of great importance, the sporadic form of AD might in future be more properly understood as a complex failure with age of multiple interacting physiological systems, some of which may share an underlying pathology [172]. For example, a strong association between the incidence of Type 2 diabetes (T2D) and AD [173] has led to a desire for a better understanding of the shared pathology of diseases which involve the aggregation of misfolded proteins and a speculation that such diseases may share complex downstream interactions. Similarly, there is increasing recognition of the role of endoplasmic reticulum (ER) stress and dysregulation of ER function in AD pathology and it has been observed that the restoration of ER stress markers by the bile acid TUDCA (tauroursodeoxycholic acid) seems to prevent AB dependent neurotoxicity in experimental models [174,175]. That the brain physiology underlying AD pathology is governed by multiple factors would hardly be surprising in an organ as functionally and structurally complex as the brain, and it is to be hoped that a greater understanding of associated physiological systems and disease states in AD will lead to further therapeutic targeting approaches out with the direct components of APP processing and AB deposition. Yet an increased understanding of the potentially multifactorial interactions of physiological systems in AD has also lent some further support to the secretase targeting approach and may even yet suggest more gentle modulatory approaches to their inhibition. For example, focus on the associations between T2D and AD led to a recognition that the impaired insulin signaling associated with AD may lead to an increase in the activity of GSK-3 [176]. GSK-3 inhibitors are recognized as a promising treatment strategy in AD due to GSK-3′s promotion of tau hyperphosphorylation and potential role in the formation of NFTs [177]. However, it has also been recognized that GSK-3 seems to regulate AB production, possibly due to its ability to interact with the presenilins and regulate the activity of the γ-secretase complex [177,178]. In addition, GSK-3 inhibition has been shown to reduce β-secretase cleavage of APP and Aβ production by decreasing BACE1 expression [178]. GSK-3 inhibition may represent a further modulatory strategy in the inhibition of secretase activity. Similarly, the strong neuroprotective effect conferred by TUDCA may be due to its inhibition of connective tissue growth factor (CTGF), a complex which enhances γ-secretase activity, raising the suggestion that the targeting of CTGF might represent a further approach in γ-secretase modulation [179].

In support of the importance of the amyloid hypothesis, the most significant high-profile discovery of recent years has been the aforementioned identification of a protective mutation in APP (A673T) in an Icelandic population which significantly reduces BACE1 cleavage of APP as compared with wild-type APP (Figure 2) [180]. This discovery was seen as providing proof of principle that reducing the amyloidogenic processing of APP has a protective effect and appeared to strongly support the idea that targeting BACE1 in the sporadic form of the disease is a justified approach. Much of the prior genetic evidence had related to the rarer early onset familial form of AD.

Further support for the amyloid hypothesis was demonstrated with the recent development of a novel three-dimensional human neural culture model of AD. This human neural stem-cell derived culture system showed that familial APP mutations induced robust Aβ deposition and led to high levels of detergent-resistant phospho-tau in the soma and neurites. In this model, inhibition of Aβ generation using β- or γ-secretase inhibitors not only reduced Aβ deposition but also attenuated the generation of aggregated phospho-tau [181]. Although this is only a single cell system, it further highlights the potential efficacy of the β- and γ-secretase inhibitors and adds weight to the hope that highly selective therapeutics with minimal off-target effects still may have potential to treat AD.

One major issue that has been highlighted by the failures of so many AD clinical trials is the design of the trials themselves. It is now generally accepted that a large number of the clinical trials of AD treatments may have failed due to the patients’ being too far advanced in the disease process to see any clinical effect from a potential therapeutic. Amyloid deposition in AD is now thought to begin many years before the appearance of cognitive symptoms and ultimate diagnosis of dementia [182]. Much drug development in AD is now beginning to focus on the targeting of patients at the very early stages of the disease, before obvious dementia, particularly in groups with familial AD. As such, the FDA have produced guidance for the design of clinical trials involving patients who do not present with overt dementia. In a *New England Journal of Medicine* (NEJM) editorial, authors of the FDA's report suggested that assessment of cognitive function might productively be combined with assessment of certain biomarkers such as levels of Aβ plaque load in the brain, measured by positron-emission tomography or levels of Aβ and tau in the cerebrospinal fluid [183]. It will be interesting to see whether some of the previously failed APP secretase targeting drugs will have clinical efficacy when executed in newly designed clinical trials.

One consideration is that if relatively healthy people are to be utilized as subjects in the drug discovery process, the prospect of ongoing problems with potential side effects could become even more serious. It is not yet clear what level of β or γ-secretase reduction may be required to achieve sufficient reduction in brain Aβ and whether this will bring with it an acceptable level of side effects. The suspension of the BACE1 inhibitor LY2886721 due to liver toxicity was initially assumed to be due to off target effects yet worryingly it was recently suggested that this effect may have come about due to BACE1's cleavage of beta-galactoside alpha 2,6-sialyltransferase (ST6Gal1) within the liver [184]. While BACE1 is expressed most highly in the brain and pancreas it is expressed at some level in all tissues and important functions may extend beyond the brain [185]. Therefore, it may be that strategies that allow for tissue-specific regulation of BACE1 should be considered in future efforts to target this protein. The components of the γ-secretase complex are also widely expressed [186]. One suggested strategy for tissue-specific regulation of the APP secretases relates to the dysfunction of the ubiquitin proteasome system seen in many neurodegenerative diseases including AD [187]. BACE1 and the PS, PEN-2 and APH-1 components of the γ-secretase complex are all thought to be turned over via the ubiquitin proteasome degradation system (UPS) [188,189], raising the attractive possibility of regulating levels of these proteins via their associations with the highly tissue and substrate specific deubiquitinating enzymes that underlie protein turnover within the UPS.

## Future perspective

There is no question of the vast unmet clinical need for AD, particularly due to the socioeconomic burden associated with caring for people afflicted with the condition. Proponents of the amyloid hypothesis argue that the clinical trial data to date has not yet adequately tested the hypothesis; it is not yet clear whether trial failure was due to ineffective late intervention, to drug side effects masking effects on cognition, or to inadequate engagement of the target secretases by the drugs, rather than to a failure of the amyloid hypothesis itself. The outcomes of current earlier intervention trials may be key to the continued focus on the amyloid hypothesis as the central tenet of AD research. Further high profile clinical failures could potentially result in the withdrawal of major pharmaceutical companies from the funding of anti-Aβ clinical trials. Any major success would doubtless be regarded as justification of the effort and resources used in the pursuit of anti-Aβ therapies over the last decade. Any success in a secretase-targeting early intervention trial which came at a cost of significant side effects in relatively healthy individuals might well lead to a renewed focus on more indirect modulatory approaches to secretase inhibition, and to a further increase in efforts to determine high quality biomarkers for the development of the disease. Despite the recent failures in clinical trials significant hope yet lies around the secretase targeting approach.

**Executive summary**

**Background**

Alzheimer's disease (AD) is the most significant disease of the aging population.
As yet there are no available drugs that can halt or even stabilize disease progression.

**The amyloid hypothesis**

The amyloid hypothesis describes the accumulation of β amyloid (Aβ) as the event that triggers AD. Aβ is a derivative of the larger amyloid precursor protein (APP).
The roles of α-, β- and γ-secretase play an essential part in this model.

**α-secretase**

ADAM10 is likely to be the most physiologically relevant α-secretase in neurons.

**Therapeutic potential of α-secretase**

Because α-secretase processing of APP involves cleavage within the Aβ peptide sequence, precluding Aβ formation, activation α-secretase is considered a potential treatment for AD.
Activation of α-secretase via associated signaling pathways may be the best approach.

**β-secretase**

BACE1 is the β-secretase enzyme, and competes with α-secretase for APP cleavage.
BACE1 cleaves APP at the N-terminal of the Aβ domain – subsequent cleavage by γ-secretase releases Aβ isoforms.

**Therapeutic potential of β-secretase**

Inhibition of the β-secretase would block Aβ production and thus represents a therapeutic strategy in AD treatment.
The past decade has seen intense research efforts toward BACE1 inhibition but as yet there have been no successes in clinical trials.
Concerns over potential side effects from this approach have grown with time and increased understanding of the role of this enzyme.
Partial inhibition of BACE1 activity could represent a feasible strategy.

**γ-secretase**

γ-secretase is a protein complex comprising presenilin, nicastrin, APH-1 and PEN-2. It cleaves the APP fragment following β-secretase cleavage to produce Aβ.
As with β-secretase, inhibition of γ-secretase represents an obvious strategy for inhibiting Aβ.

**Therapeutic potential of γ-secretase**

Clinical trials of γ-secretase inhibitors have demonstrated significant side effects and lack of positive effect on cognition. This is probably because γ-secretase cleaves a number of important substrates other than APP.
Hope remains around two newer strategies which aim to avoid an important second substrate of γ-secretase called Notch.

**Conclusion**

It remains far from certain whether targeting the secretases involved in APP processing will yield therapeutic success.
High profile clinical failures have called into question the viability of the amyloid hypothesis.
A number of the clinical trials of AD treatments may have failed due to the patients’ being too far advanced in the in the disease process. Newer trials will target patients at earlier stages of the disease.
The outcomes of such trials may be key to continued focus on the amyloid hypothesis as the central tenet of AD research.
